# Second-order lower radial tangent derivatives and applications to set-valued optimization

**DOI:** 10.1186/s13660-016-1275-x

**Published:** 2017-01-04

**Authors:** Bihang Xu, Zhenhua Peng, Yihong Xu

**Affiliations:** 1 School of Information Engineering, Nanchang University, Nanchang, 330031 China; 2 School of Mathematics and Statistics, Wuhan University, Wuhan, 430072 China; 3 Department of Mathematics, Nanchang University, Nanchang, 330031 China

**Keywords:** 46G05, 90C29, 90C46, Henig efficiency, radial tangent derivative, set-valued optimization, optimality condition

## Abstract

We introduce the concepts of second-order radial composed tangent derivative, second-order radial tangent derivative, second-order lower radial composed tangent derivative, and second-order lower radial tangent derivative for set-valued maps by means of a radial tangent cone, second-order radial tangent set, lower radial tangent cone, and second-order lower radial tangent set, respectively. Some properties of second-order tangent derivatives are discussed, using which second-order necessary optimality conditions are established for a point pair to be a Henig efficient element of a set-valued optimization problem, and in the expressions the second-order tangent derivatives of the objective function and the constraint function are separated.

## Introduction

In recent years, first-order optimality conditions in the set-valued optimization have attracted a great deal of attention, and various derivative-like notions have been utilized to express these optimality conditions. For example, Gong *et al.* [1] introduced the concept of radial tangent cone and presented several kinds of necessary and sufficient conditions for some proper efficiencies, Kasimbeyli [2] gave necessary and sufficient optimality conditions based on the concept of the radial epiderivatives. At the same time, second-order optimality conditions and higher-order optimality conditions for vector optimization problems have been extensively studied in the literature (see [3–18]). Jahn *et al.* [3] proposed second-order epiderivatives for set-valued maps, and by using these concepts they gave second-order necessary optimality conditions and a sufficient optimality condition in set optimization. Khan and Isac [15] proposed the concept of a second-order composed contingent derivative for set-valued maps, using which they established second-order optimality conditions in set-valued optimization. With a second-order composed contingent derivative, Zhu *et al.* [4] established second-order Karush-Kuhn-Tucker necessary and sufficient optimality conditions for a set-valued optimization problem. However, in [1, 4, 7–12, 14], in the expressions of first-order and higher-order optimality conditions, the tangent derivatives of the objective function and the constraint function are not separated, and thus the properties of the derivatives of the objective function are not easily obtained from those of the constraint function.

On the other hand, some efficient points exhibit certain abnormal properties. To eliminate such anomalous efficient points, various concepts of proper efficiency have been introduced [19–22]. Henig [19] introduced the concept of Henig efficiency, which is very important for the study of set-valued optimization [13, 14, 20, 23].

In this paper, we introduce a new class of lower radial tangent cones and two new kinds of second-order tangent sets, using which we introduce four new kinds of second-order tangent derivatives. We discuss the properties of these second-order tangent derivatives, using which we establish second-order necessary optimality conditions for a point pair to be a Henig efficient element of a set-valued optimization problem.

## Basic concepts

Throughout the paper, let *X*, *Y*, and *Z* be three real normed linear spaces, $0_{X}$, $0_{Y}$, and $0_{Z}$ denote the original points of *X*, *Y*, and *Z*, respectively. Let *M* be a nonempty subset of *Y*. As usual, we denote the interior, closure, and cone hull of *M* by int*M*, cl*M*, and cone*M*, respectively. The cone hull of *M* is defined by

$$\operatorname{cone}M=\{\lambda m:\lambda\geq0, m\in M\}. $$

Let *C* and *D* be closed convex pointed cones in *Y* and *Z*, respectively. A nonempty convex subset $B\subset C$ is called a base of *C* if $0\notin\operatorname{cl}B$ and $C=\operatorname{cone}B$.

Denote the closed unit ball of *Y* by *U*. Suppose that *C* has a base *B*. Let $\delta:=\inf\{\|b\|:b\in B\}$ and

$$C_{\varepsilon}(B):=\operatorname{cone}(\varepsilon U+B) $$

for all $0<\varepsilon<\delta$. It is clear that $\delta>0$ and $C_{\varepsilon}(B)$ is a pointed convex cone for all $0<\varepsilon <\delta$ (see [21]).

Let $F:X\rightarrow2^{Y}$ be a set-valued map. The domain, graph, and epigraph of *F* are defined respectively by

$$\begin{aligned}& \operatorname{dom}F:=\bigl\{ x\in X:F(x)\neq\emptyset\bigr\} , \\& \operatorname{graph}F:=\bigl\{ (x,y)\in X\times Y:y\in F(x) \bigr\} , \\& \operatorname{epi}F:=\bigl\{ (x,y)\in X\times Y:y\in F(x)+C \bigr\} . \end{aligned}$$

### Definition 2.1

See [1]

Let *A* be a nonempty subset of *X*, and let $\hat{x}\in\operatorname{cl}A$. The radial tangent cone of *A* at *x̂*, denoted by $R(A,\hat{x})$, is given by

2.1 $$ R(A,\hat{x}):=\bigl\{ u\in X: \exists t_{n}>0 \mbox{ and } x_{n}\in A \mbox{ such that } t_{n}(x_{n}- \hat{x})\rightarrow u\bigr\} . $$

### Remark 2.1

Equation (2.1) is equivalent to

$$R(A,\hat{x})=\{u\in X: \exists\lambda_{n}>0\mbox{ and } u_{n}\rightarrow u \mbox{ such that } \hat{x}+\lambda_{n}u_{n} \in A, \forall n\in N\}, $$

where *N* denotes the set of positive integers.

### Definition 2.2

See [24]

Let *A* be a nonempty subset of *X*, and let $\hat{x}\in\operatorname{cl}A$. The contingent cone of *A* at *x̂*, denoted by $T(A,\hat{x})$, is given by

2.2 $$ T(A,\hat{x}):=\bigl\{ u\in X: \exists t_{n}\rightarrow0^{+} \mbox{ and } u_{n}\rightarrow u \mbox{ such that } \hat{x}+t_{n}u_{n}\in A, \forall n\in N\bigr\} . $$

### Remark 2.2

See [24]

Equation (2.2) is equivalent to

$$T(A,\hat{x}):=\bigl\{ u\in X: \exists\lambda_{n}\rightarrow+\infty \mbox{ and } x_{n}\in A \mbox{ such that } x_{n}\rightarrow \hat{x} \mbox{ and } \lambda_{n}(x_{n}-\hat{x})\rightarrow u \bigr\} . $$

### Definition 2.3

See [3]

Let *A* be a nonempty subset of *X*, and let $\hat{x}\in\operatorname{cl}A$. The second-order contingent set of *A* at *x̂* in the direction *w*, denoted by $T^{2}(A,\hat {x},w)$, is given by

$$T^{2}(A,\hat{x},w):=\biggl\{ v\in X: \exists t_{n} \rightarrow0^{+} \mbox{ and } v_{n}\rightarrow v \mbox{ such that } \hat{x}+t_{n}w+\frac {1}{2}t_{n}^{2}v_{n} \in A\biggr\} . $$

### Definition 2.4

See [4, 15]

Let $F:X\rightarrow2^{Y}$ be a set-valued map, $(\hat{x},\hat{y})\in\operatorname{graph}F$, and $(\hat{u},\hat{v})\in X\times Y$. The second-order composed contingent derivative of *F* at $(\hat{x},\hat{y})$ in the direction $(\hat{u},\hat{v})$ is the set-valued map $D^{\prime\prime}F(\hat {x},\hat{y},\hat{u},\hat{v}) :X\rightarrow2^{Y}$ defined by

$$\operatorname{graph}D^{\prime\prime}F(\hat{x},\hat{y},\hat{u},\hat {v})=T\bigl(T \bigl(\operatorname{graph}F,(\hat{x},\hat{y})\bigr),(\hat{u},\hat{v})\bigr). $$

### Definition 2.5

See [3]

Let $F:X\rightarrow2^{Y}$ be a set-valued map, $(\hat{x},\hat{y})\in\operatorname{graph}F$, and $(\hat{u},\hat{v})\in X\times Y$. The second-order contingent derivative of *F* at $(\hat{x},\hat{y})$ in the direction $(\hat{u},\hat{v})$ is the set-valued map $D^{2}F(\hat{x},\hat{y},\hat {u},\hat{v}) :X\rightarrow2^{Y}$ defined by

$$D^{2}F(\hat{x},\hat{y},\hat{u},\hat{v}) (x)=\bigl\{ y\in Y:(x,y)\in T^{2}\bigl(\operatorname{graph}F,(\hat{x},\hat{y}),(\hat{u},\hat{v}) \bigr)\bigr\} . $$

In the following, we introduce a new class of lower radial tangent cones and two new kinds of second-order tangent sets.

### Definition 2.6

Let *Q* be a nonempty subset of $X\times Y$, and let $(\hat{x},\hat{y})\in\operatorname{cl}Q$. The lower radial tangent cone of *Q* at $(\hat{x},\hat{y})$ is defined by

$$\begin{aligned} R_{l}\bigl(Q,(\hat{x},\hat{y})\bigr) :=&\bigl\{ (u,v)\in X\times Y: \forall t_{n}>0, \forall u_{n}\rightarrow u, \exists v_{n}\rightarrow v \\ & \mbox{such that } (\hat{x}+t_{n}u_{n}, \hat{y}+t_{n}v_{n})\in Q\bigr\} . \end{aligned}$$

### Definition 2.7

Let *Q* be a nonempty subset of $X\times Y$, and let $(\hat{x},\hat{y})\in\operatorname{cl}Q$. The second-order lower radial tangent set of *Q* at $(\hat{x},\hat{y})$ in the direction $(\hat{u},\hat{v})$, denoted by $R_{l}^{2}(Q,(\hat{x},\hat{y}), (\hat {u},\hat{v}))$, is given by

$$\begin{aligned} R_{l}^{2}\bigl(Q,(\hat{x},\hat{y}),(\hat{u},\hat{v}) \bigr) :=&\biggl\{ (u,v)\in X\times Y: \forall t_{n}>0, \forall u_{n}\rightarrow u, \exists v_{n}\rightarrow v \\ & \mbox{such that } \biggl(\hat{x}+t_{n}\hat{u}+ \frac {1}{2}t_{n}^{2}u_{n}, \hat{y}+t_{n}\hat{v}+\frac{1}{2}t_{n}^{2}v_{n} \biggr)\in Q\biggr\} . \end{aligned}$$

### Definition 2.8

Let *A* be a nonempty subset of *X*, and let $\hat{x}\in\operatorname{cl}A$. The second-order radial tangent set of *A* at *x̂* in the direction *w*, denoted by $R^{2}(A,\hat{x},w)$, is given by

$$R^{2}(A,\hat{x},w):=\biggl\{ v\in X: \exists t_{n}>0\mbox{ and } v_{n}\rightarrow v \mbox{ such that } \hat{x}+t_{n}w+ \frac {1}{2}t_{n}^{2}v_{n}\in A\biggr\} . $$

### Remark 2.3

Let $\emptyset\neq Q\subset X\times Y$, $(\hat {x},\hat{y})\in\operatorname{cl}Q$. Then

(i) $R_{l}(Q,(\hat{x},\hat{y}))\subset T(Q,(\hat{x},\hat {y}))\subset R(Q,(\hat{x},\hat{y}))$;
(ii) $R_{l}^{2}(Q,(\hat{x},\hat{y}),(\hat{u},\hat{v}))\subset T^{2}(Q,(\hat{x},\hat{y}),(\hat{u},\hat{v}))\subset R^{2}(Q,(\hat {x},\hat{y}),(\hat{u},\hat{v}))$.

However, none of the inverse inclusions is necessarily true, as is shown in the following example.

### Example 2.1

Let *R* be the set of real numbers, $X=Y=R$, $Q=\{(-\frac{1}{n},\frac{1}{n^{2}}):n=1,2,\ldots\}\cup\{(x,y):x\geq 0,y\geq0\}\cup\{(-1,-1)\}$, and $(\hat{x},\hat{y})=(\hat{u},\hat{v})=(0,0)$. A direct calculation gives $R_{l}^{2}(Q,(0,0),(0,0))=\{(x,y):x>0,y\geq0\}$, $T^{2}(Q,(0,0),(0,0))=\{(x,y):x\geq0,y\geq0\}\cup\{(x,0):x<0\}$, and $R^{2}(Q,(0,0),(0,0))=\{(x,y):x\geq0,y\geq0\}\cup\{(x,0):x<0\}\cup\{ (x,x):x<0\}\cup\bigcup_{n=1}^{\infty}\{\lambda(-\frac{1}{n},\frac {1}{n^{2}}):\lambda>0\}$.

## The second-order lower radial tangent derivative

In this section, by virtue of the radial tangent cone, the second-order radial tangent set, the lower radial tangent cone, and the second-order lower radial tangent set, we introduce the concepts of the second-order radial composed tangent derivative, the second-order radial tangent derivative, the second-order lower radial composed tangent derivative, and the second-order lower radial tangent derivative for a set-valued map. Furthermore, we discuss some important properties of the second-order lower radial composed tangent derivative and the second-order lower radial tangent derivative.

### Definition 3.1

Let $F:X\rightarrow2^{Y}$ be a set-valued map, $(\hat{x},\hat{y})\in\operatorname{graph}F$, and $(\hat{u},\hat {v})\in X\times Y$. The second-order radial composed tangent derivative of *F* at $(\hat{x},\hat{y})$ in the direction $(\hat{u},\hat{v})$ is the set-valued map $R^{\prime\prime}F(\hat {x},\hat{y},\hat{u},\hat{v}) :X\rightarrow2^{Y}$ defined by

$$\operatorname{graph}R^{\prime\prime}F(\hat{x},\hat{y},\hat{u},\hat {v})=R\bigl(R \bigl(\operatorname{epi}F,(\hat{x},\hat{y})\bigr),(\hat{u},\hat{v})\bigr). $$

If $R(R(\operatorname{epi}F,(\hat{x},\hat{y})),(\hat{u},\hat{v}))\neq \emptyset$, then *F* is said to be second-order radial composed derivable at $(\hat{x},\hat{y})$ in the direction $(\hat{u},\hat{v})$ or that the second-order radial composed tangent derivative of *F* at $(\hat{x},\hat{y})$ in the direction $(\hat{u},\hat{v})$ exists.

### Definition 3.2

Let $F:X\rightarrow2^{Y}$ be a set-valued map, $(\hat{x},\hat{y})\in\operatorname{graph}F$, and $(\hat{u},\hat{v})\in X\times Y$. The second-order radial tangent derivative of *F* at $(\hat{x},\hat{y})$ in the direction $(\hat{u},\hat{v})$ is the set-valued map $R^{2}F(\hat{x},\hat{y},\hat {u},\hat{v}) :X\rightarrow2^{Y}$ defined by

$$\operatorname{graph}R^{2}F(\hat{x},\hat{y},\hat{u},\hat {v})=R^{2}\bigl(\operatorname{epi}F,(\hat{x},\hat{y}),(\hat{u},\hat{v}) \bigr). $$

If $R^{2}(\operatorname{epi}F,(\hat{x},\hat{y}),(\hat{u},\hat{v}))\neq \emptyset$, then *F* is called second-order radial derivable at $(\hat{x},\hat{y})$ in the direction $(\hat{u},\hat{v})$ or that the second-order radial tangent derivative of *F* at $(\hat{x},\hat{y})$ in the direction $(\hat{u},\hat{v})$ exists.

### Definition 3.3

Let $F:X\rightarrow2^{Y}$ be a set-valued map, $(\hat{x},\hat{y})\in\operatorname{graph}F$, and $(\hat{u},\hat{v})\in X\times Y$. The second-order lower radial composed tangent derivative of *F* at $(\hat{x},\hat{y})$ in the direction $(\hat{u},\hat{v})$ is the set-valued map $R_{l}^{\prime\prime}F(\hat {x},\hat{y},\hat{u},\hat{v}) :X\rightarrow2^{Y}$ defined by

$$\operatorname{graph}R_{l}^{\prime\prime}F(\hat{x},\hat{y},\hat{u},\hat {v})=R_{l}\bigl(R_{l}\bigl(\operatorname{epi}F,(\hat{x}, \hat{y})\bigr),(\hat{u},\hat{v})\bigr). $$

If $R_{l}(R_{l}(\operatorname{epi}F,(\hat{x},\hat{y})),(\hat{u},\hat {v}))\neq\emptyset$, then *F* is said to be second-order lower radial composed derivable at $(\hat{x},\hat{y})$ in the direction $(\hat{u},\hat{v})$ or that the second-order lower radial composed tangent derivative of *F* at $(\hat{x},\hat{y})$ in the direction $(\hat{u},\hat{v})$ exists.

### Definition 3.4

Let $F:X\rightarrow2^{Y}$ be a set-valued map, $(\hat{x},\hat{y})\in\operatorname{graph}F$, and $(\hat{u},\hat{v})\in X\times Y$. The second-order lower radial tangent derivative of *F* at $(\hat{x},\hat{y})$ in the direction $(\hat{u},\hat{v})$ is the set-valued map $R_{l}^{2}F(\hat{x},\hat {y},\hat{u},\hat{v}) :X\rightarrow2^{Y}$ defined by

$$\operatorname{graph}R_{l}^{2}F(\hat{x},\hat{y},\hat{u},\hat {v})=R_{l}^{2}\bigl(\operatorname{epi}F,(\hat{x},\hat{y}),( \hat{u},\hat{v})\bigr). $$

If $R_{l}^{2}(\operatorname{epi}F,(\hat{x},\hat{y}),(\hat{u},\hat {v}))\neq\emptyset$, then *F* is called second-order lower radial derivable at $(\hat{x},\hat{y})$ in the direction $(\hat{u},\hat{v})$ or that the second-order lower radial tangent derivative of *F* at $(\hat{x},\hat{y})$ in the direction $(\hat{u},\hat{v})$ exists.

### Proposition 3.1

*Suppose that* $E\subset X$ *and the second*-*order lower radial composed tangent derivative of* $F:X\rightarrow2^{Y}$ *at* $(\hat{x},\hat{y})\in\operatorname{graph}F$ *in the direction* $(\hat {u},\hat{v})$ *exists*. *Then*

$$R_{l}^{\prime\prime}F(\hat{x},\hat{y},\hat{u},\hat{v}) \bigl(R \bigl(R(E,\hat {x}),\hat{u}\bigr) \bigr)\subset\operatorname{clcone} \bigl( \operatorname{clcone}\bigl(F(E)+C-\hat{y}\bigr)-\hat{v} \bigr). $$

### Proof

Let $v\in R_{l}^{\prime\prime}F(\hat{x},\hat{y},\hat {u},\hat{v}) (R(R(E,\hat{x}),\hat{u}) )$. Then there exists $u\in R(R(E,\hat{x}),\hat{u})$ such that

$$v\in R_{l}^{\prime\prime}F(\hat{x},\hat{y},\hat{u},\hat{v}) (u). $$

Thus,

3.1 $$ (u,v)\in\operatorname{graph}R_{l}^{\prime\prime}F(\hat{x},\hat{y},\hat {u},\hat{v})=R_{l}\bigl(R_{l}\bigl(\operatorname{epi}F,( \hat{x},\hat{y})\bigr),(\hat {u},\hat{v})\bigr). $$

From $u\in R(R(E,\hat{x}),\hat{u})$ it follows that there exist sequences $t_{n}>0$ and $u_{n}\rightarrow u$ such that

$$\hat{u}+t_{n}u_{n}\in R(E,\hat{x}). $$

Therefore, there exist sequences $t_{n}^{k}>0$ and $u_{n}^{k}\rightarrow\hat{u}+t_{n}u_{n}$ such that

$$\hat{x}+t_{n}^{k}u_{n}^{k}\in E. $$

For such $t_{n}$ and $u_{n}$, it follows from (3.1) that there exists a sequence $v_{n}\rightarrow v$ such that

$$(\hat{u}+t_{n}u_{n}, \hat{v}+t_{n}v_{n}) \in R_{l}\bigl(\operatorname {epi}F,(\hat{x},\hat{y})\bigr). $$

Then, for the same $t_{n}^{k}$ and $u_{n}^{k}$, there exists a sequence $v_{n}^{k}\rightarrow\hat{v}+t_{n}v_{n}$ such that

$$\bigl(\hat{x}+t_{n}^{k}u_{n}^{k}, \hat{y}+t_{n}^{k}v_{n}^{k}\bigr)\in \operatorname{epi}F, $$

and, consequently,

$$\hat{y}+t_{n}^{k}v_{n}^{k}\in F\bigl( \hat{x}+t_{n}^{k}u_{n}^{k}\bigr)+C. $$

Thus,

$$v_{n}^{k}\in\frac{1}{t_{n}^{k}} \bigl(F\bigl(\hat {x}+t_{n}^{k}u_{n}^{k}\bigr)+C-\hat{y} \bigr), $$

and, consequently,

$$v_{n}^{k}\in\operatorname{cone} \bigl(F(E)+C-\hat{y} \bigr). $$

Since $v_{n}^{k}\rightarrow\hat{v}+t_{n}v_{n}$ as $k\rightarrow\infty $, we obtain

$$\hat{v}+t_{n}v_{n}\in\operatorname{clcone} \bigl(F(E)+C- \hat{y} \bigr). $$

Thus,

$$v_{n}\in\frac{1}{t_{n}} \bigl(\operatorname{clcone} \bigl(F(E)+C- \hat {y} \bigr)-\hat{v} \bigr), $$

and, consequently,

$$v_{n}\in\operatorname{cone} \bigl(\operatorname{clcone} \bigl(F(E)+C- \hat {y} \bigr)-\hat{v} \bigr). $$

Taking $n\rightarrow\infty$, we get

$$v\in\operatorname{clcone} \bigl(\operatorname{clcone} \bigl(F(E)+C-\hat {y} \bigr)-\hat{v} \bigr). $$

So,

$$R_{l}^{\prime\prime}F(\hat{x},\hat{y},\hat{u},\hat{v}) \bigl(R \bigl(R(E,\hat {x}),\hat{u}\bigr) \bigr)\subset\operatorname{clcone} \bigl( \operatorname{clcone}\bigl(F(E)+C-\hat{y}\bigr)-\hat{v} \bigr). $$

 □

### Proposition 3.2

*Suppose that* $E\subset X$ *and the second*-*order lower radial tangent derivative of* $F:X\rightarrow2^{Y}$ *at* $(\hat{x},\hat{y})\in\operatorname{graph}F$ *in the direction* $(\hat {u},\hat{v})$ *exists*. *Then*

$$R_{l}^{2}F(\hat{x},\hat{y},\hat{u},\hat{v}) \bigl(R^{2}(E,\hat{x},\hat {u}) \bigr)\subset\operatorname{clcone} \bigl(\operatorname{cone}\bigl(F(E)+C-\hat{y}\bigr)-\hat{v} \bigr). $$

### Proof

Let $v\in R_{l}^{2}F(\hat{x},\hat{y},\hat{u},\hat {v}) (R^{2}(E,\hat{x},\hat{u}) )$. Then there exists $u\in R^{2}(E,\hat{x},\hat{u})$ such that

$$v\in R_{l}^{2}F(\hat{x},\hat{y},\hat{u},\hat{v}) (u). $$

Thus,

3.2 $$ (u,v)\in\operatorname{graph}R_{l}^{2}F(\hat{x},\hat{y}, \hat{u},\hat {v})=R_{l}^{2}\bigl(\operatorname{epi}F,( \hat{x},\hat{y}),(\hat{u},\hat {v})\bigr). $$

From $u\in R^{2}(E,\hat{x},\hat{u})$ it follows that there exist sequences $t_{n}>0$ and $u_{n}\rightarrow u$ such that

$$\hat{x}+t_{n}\hat{u}+\frac{1}{2}t_{n}^{2}u_{n} \in E. $$

For such $t_{n}$ and $u_{n}$, it follows from (3.2) that there exists a sequence $v_{n}\rightarrow v$ such that

$$\biggl(\hat{x}+t_{n}\hat{u}+\frac{1}{2}t_{n}^{2}u_{n}, \hat{y}+t_{n}\hat {v}+\frac{1}{2}t_{n}^{2}v_{n} \biggr)\in\operatorname{epi}F. $$

Then

$$\hat{y}+t_{n}\hat{v}+\frac{1}{2}t_{n}^{2}v_{n} \in F\biggl(\hat{x}+t_{n}\hat {u}+\frac{1}{2}t_{n}^{2}u_{n} \biggr)+C, $$

and, consequently,

$$\hat{v}+\frac{1}{2}t_{n}v_{n}\in\frac{1}{t_{n}} \biggl(F\biggl(\hat {x}+t_{n}\hat{u}+\frac{1}{2}t_{n}^{2}u_{n} \biggr)+C-\hat{y} \biggr). $$

Thus,

$$\hat{v}+\frac{1}{2}t_{n}v_{n}\in\operatorname{cone} \bigl(F(E)+C-\hat {y} \bigr). $$

Hence,

$$v_{n}\in\frac{2}{t_{n}} \bigl(\operatorname{cone} \bigl(F(E)+C-\hat {y} \bigr)-\hat{v} \bigr). $$

Therefore,

$$v_{n}\in\operatorname{cone} \bigl(\operatorname{cone} \bigl(F(E)+C- \hat {y} \bigr)-\hat{v} \bigr). $$

Taking $n\rightarrow\infty$, we get

$$v\in\operatorname{clcone} \bigl(\operatorname{cone} \bigl(F(E)+C-\hat {y} \bigr)-\hat{v} \bigr). $$

So,

$$R_{l}^{2}F(\hat{x},\hat{y},\hat{u},\hat{v}) \bigl(R^{2}(E,\hat{x},\hat {u}) \bigr)\subset\operatorname{clcone} \bigl(\operatorname{cone}\bigl(F(E)+C-\hat{y}\bigr)-\hat{v} \bigr). $$

 □

### Remark 3.1

If we substitute $D^{\prime\prime}F(\hat{x},\hat {y},\hat{u},\hat{v})$ or $R^{\prime\prime}F(\hat{x},\hat{y},\hat{u},\hat {v})$ for $R_{l}^{\prime\prime}F(\hat{x},\hat{y},\hat{u},\hat{v})$ in Proposition 3.1, then none of the inclusions

$$D^{\prime\prime}F(\hat{x},\hat{y},\hat{u},\hat{v}) \bigl(R\bigl(R(E,\hat {x}), \hat{u}\bigr) \bigr)\subset\operatorname{clcone} \bigl(\operatorname{clcone} \bigl(F(E)+C-\hat{y}\bigr)-\hat{v} \bigr) $$

and

$$R^{\prime\prime}F(\hat{x},\hat{y},\hat{u},\hat{v}) \bigl(R\bigl(R(E,\hat {x}), \hat{u}\bigr) \bigr)\subset\operatorname{clcone} \bigl(\operatorname {clcone} \bigl(F(E)+C-\hat{y}\bigr)-\hat{v} \bigr) $$

is necessarily true. If we substitute $D^{2}F(\hat{x},\hat{y},\hat {u},\hat{v})$ or $R^{2}F(\hat{x},\hat{y},\hat{u},\hat{v})$ for $R_{l}^{2}F(\hat{x},\hat{y},\hat{u},\hat{v})$ in Proposition 3.2, then none of the inclusions

$$D^{2}F(\hat{x},\hat{y},\hat{u},\hat{v}) \bigl(R^{2}(E, \hat{x},\hat {u}) \bigr)\subset\operatorname{clcone} \bigl(\operatorname {cone} \bigl(F(E)+C-\hat{y}\bigr)-\hat{v} \bigr) $$

and

$$R^{2}F(\hat{x},\hat{y},\hat{u},\hat{v}) \bigl(R^{2}(E, \hat{x},\hat {u}) \bigr)\subset\operatorname{clcone} \bigl(\operatorname {cone} \bigl(F(E)+C-\hat{y}\bigr)-\hat{v} \bigr) $$

is necessarily true, as is shown in the following example.

### Example 3.1

Let *R* be the set of real numbers, $X=Y=R$, $C=\{t:t\geq0\}$, and $E=\{x:x\geq0\}$. Define the set-valued map $F:X\rightarrow2^{Y}$ by

$$F(x)=\left \{ \textstyle\begin{array}{l@{\quad}l} \{y:y\geq0\}& \mbox{if } x\geq0, \\ \{y:y\geq\sqrt[3]{x}\} & \mbox{otherwise}. \end{array}\displaystyle \right . $$

(i) Let $(\hat{x},\hat{y})=(0,0)$, $(\hat{u},\hat{v})=(0,-1)$. A direct calculation gives

$$\begin{aligned}& R(E,0)=R\bigl(R(E,0),0\bigr)=[0,+\infty), \\& T\bigl(\operatorname{epi}F,(0,0)\bigr)=R\bigl(\operatorname{epi}F,(0,0)\bigr)= \bigl\{ (x,y):x>0,y\geq0\bigr\} \cup\bigl\{ (x,y):x\leq0,y\in R\bigr\} , \\& T \bigl(T\bigl(\operatorname{epi}F,(0,0)\bigr),(0,-1) \bigr)=\bigl\{ (x,y):x \leq0,y\in R\bigr\} , \\& R \bigl(R\bigl(\operatorname{epi}F,(0,0)\bigr),(0,-1) \bigr)=\bigl\{ (x,y):x \leq0,y\in R\bigr\} \cup\bigl\{ (x,y):x>0,y\geq0\bigr\} , \\& D^{\prime\prime}F(0,0,0,-1) (x)=\left \{ \textstyle\begin{array}{l@{\quad}l} R,& x\leq0, \\ \emptyset, & x>0, \end{array}\displaystyle \right . \\& R^{\prime\prime}F(0,0,0,-1) (x)=\left \{ \textstyle\begin{array}{l@{\quad}l} R,& x\leq0, \\ \{y:y\geq0\}, & x>0, \end{array}\displaystyle \right . \\& R_{l}\bigl(\operatorname{epi}F,(0,0)\bigr)=\bigl\{ (x,y):x\in R,y\geq0 \bigr\} , \\& R_{l} \bigl(R_{l}\bigl(\operatorname{epi}F,(0,0) \bigr),(0,-1) \bigr)=\emptyset, \\& R_{l}^{\prime\prime}F(0,0,0,-1) (x)=\emptyset,\quad x\in R. \end{aligned}$$

Consequently,

$$\begin{aligned}& D^{\prime\prime}F(0,0,0,-1) \bigl(R\bigl(R(E,0),0\bigr)\bigr)=R^{\prime\prime }F(0,0,0,-1) \bigl(R\bigl(R(E,0),0\bigr)\bigr)=R, \\& R_{l}^{\prime\prime}F(0,0,0,-1) \bigl(R\bigl(R(E,0),0\bigr)\bigr)= \emptyset, \\& \operatorname{clcone} \bigl(\operatorname{clcone}\bigl(F(E)+C-\hat{y}\bigr)-\hat {v} \bigr)=[0,+\infty). \end{aligned}$$

Then, the inclusion of Proposition 3.1 is true. However,

$$D^{\prime\prime}F(\hat{x},\hat{y},\hat{u},\hat{v}) \bigl(R\bigl(R(E,\hat {x}), \hat{u}\bigr) \bigr)\not\subset\operatorname{clcone} \bigl(\operatorname{clcone} \bigl(F(E)+C-\hat{y}\bigr)-\hat{v} \bigr) $$

and

$$R^{\prime\prime}F(\hat{x},\hat{y},\hat{u},\hat{v}) \bigl(R\bigl(R(E,\hat {x}), \hat{u}\bigr) \bigr)\not\subset\operatorname{clcone} \bigl(\operatorname{clcone} \bigl(F(E)+C-\hat{y}\bigr)-\hat{v} \bigr). $$

(ii) Let $(\hat{x},\hat{y})=(0,0)$, $(\hat{u},\hat{v})=(0,0)$. A direct calculation gives

$$\begin{aligned}& R\bigl(R(E,0),0\bigr)=R^{2}(E,0,0)=R(E,0)=[0,+\infty), \\& T \bigl(T\bigl(\operatorname{epi}F,(0,0)\bigr),(0,0) \bigr)=R \bigl(R\bigl( \operatorname{epi}F,(0,0)\bigr),(0,0) \bigr)=T^{2}\bigl(\operatorname {epi}F,(0,0),(0,0)\bigr) \\& \hphantom{T \bigl(T\bigl(\operatorname{epi}F,(0,0)\bigr),(0,0) \bigr)} =R^{2}\bigl(\operatorname{epi}F,(0,0),(0,0)\bigr) \\& \hphantom{T \bigl(T\bigl(\operatorname{epi}F,(0,0)\bigr),(0,0) \bigr)}=T \bigl(\operatorname {epi}F,(0,0)\bigr)=R\bigl(\operatorname{epi}F,(0,0)\bigr) \\& \hphantom{T \bigl(T\bigl(\operatorname{epi}F,(0,0)\bigr),(0,0) \bigr)} =\bigl\{ (x,y):x>0,y\geq0\bigr\} \cup\bigl\{ (x,y):x\leq0,y\in R \bigr\} , \\& D^{\prime\prime}F(0,0,0,0) (x)=R^{\prime\prime}F(0,0,0,0) (x)=\left \{ \textstyle\begin{array}{l@{\quad}l} R,& x\leq0, \\ \{y:y\geq0\}, & x>0, \end{array}\displaystyle \right . \\& D^{2}F(0,0,0,0) (x)=R^{2}F(0,0,0,0) (x)=\left \{ \textstyle\begin{array}{l@{\quad}l} R,& x\leq0, \\ \{y:y\geq0\}, & x>0, \end{array}\displaystyle \right . \\& R_{l} \bigl(R_{l}\bigl(\operatorname {epi}F,(0,0) \bigr),(0,0) \bigr)=R_{l}^{2}\bigl(\operatorname{epi}F,(0,0),(0,0) \bigr) \\& \hphantom{R_{l} \bigl(R_{l}\bigl(\operatorname {epi}F,(0,0) \bigr),(0,0) \bigr)} =R_{l}\bigl(\operatorname{epi}F,(0,0)\bigr)=\bigl\{ (x,y):x\in R,y\geq0\bigr\} , \\& R_{l}^{\prime\prime}F(0,0,0,0) (x)=R_{l}^{2}F(0,0,0,0) (x)=[0,+\infty ),\quad x\in R. \end{aligned}$$

Consequently,

$$\begin{aligned}& D^{\prime\prime}F(0,0,0,0) \bigl(R\bigl(R(E,0),0\bigr)\bigr)=R^{\prime \prime}F(0,0,0,0) \bigl(R\bigl(R(E,0),0\bigr)\bigr) \\& \hphantom{D^{\prime\prime}F(0,0,0,0) \bigl(R\bigl(R(E,0),0\bigr)\bigr)}=D^{2}F(0,0,0,0) \bigl(R^{2}(E,0,0) \bigr) \\& \hphantom{D^{\prime\prime}F(0,0,0,0) \bigl(R\bigl(R(E,0),0\bigr)\bigr)}=R^{2}F(0,0,0,0) \bigl(R^{2}(E,0,0)\bigr)=R, \\& R_{l}^{\prime\prime }F(0,0,0,0) \bigl(R\bigl(R(E,0),0\bigr) \bigr)=R_{l}^{2}F(0,0,0,0) \bigl(R^{2}(E,0,0) \bigr)=[0,+\infty), \\& \operatorname{clcone} \bigl(\operatorname{clcone}\bigl(F(E)+C-\hat{y}\bigr)-\hat {v} \bigr)=\operatorname{clcone} \bigl(\operatorname{cone}\bigl(F(E)+C-\hat {y} \bigr)-\hat{v} \bigr)=[0,+\infty). \end{aligned}$$

Then, the inclusions of Propositions 3.1 and 3.2 are true. However,

$$\begin{aligned}& D^{\prime\prime}F(\hat{x},\hat{y},\hat{u},\hat{v}) \bigl(R\bigl(R(E,\hat {x}), \hat{u}\bigr) \bigr)\not\subset\operatorname{clcone} \bigl(\operatorname{clcone} \bigl(F(E)+C-\hat{y}\bigr)-\hat{v} \bigr), \\& R^{\prime\prime}F(\hat{x},\hat{y},\hat{u},\hat{v}) \bigl(R\bigl(R(E,\hat {x}), \hat{u}\bigr) \bigr)\not\subset\operatorname{clcone} \bigl(\operatorname{clcone} \bigl(F(E)+C-\hat{y}\bigr)-\hat{v} \bigr), \\& D^{2}F(\hat{x},\hat{y},\hat{u},\hat{v}) \bigl(R^{2}(E, \hat{x},\hat {u}) \bigr)\not\subset\operatorname{clcone} \bigl(\operatorname {cone}\bigl(F(E)+C-\hat{y}\bigr)-\hat{v} \bigr), \end{aligned}$$

and

$$R^{2}F(\hat{x},\hat{y},\hat{u},\hat{v}) \bigl(R^{2}(E, \hat{x},\hat {u}) \bigr)\not\subset\operatorname{clcone} \bigl(\operatorname {cone}\bigl(F(E)+C-\hat{y}\bigr)-\hat{v} \bigr). $$

## Second-order necessary optimality conditions

Let $F:X\rightarrow2^{Y}$, $G:X\rightarrow2^{Z}$, and $(F,G):X\rightarrow2^{Y\times Z}$ be defined by $(F,G)(x)=F(x)\times G(x)$.

Consider the following optimization problem with set-valued maps:

$$\begin{aligned} (\mathrm{VP})&\quad \min F(x), \\ &\qquad \mbox{s.t. } G(x)\cap(-D)\neq\emptyset,\quad x\in X. \end{aligned}$$

The feasible set of (VP) is denoted by *Ê*, that is, $\hat{E}=\{ x\in X:G(x)\cap(-D)\neq\emptyset\}$.

### Definition 4.1

See [13, 19, 21]

Let $\hat{x}\in\hat{E}$, $\hat{y}\in F(\hat{x})$. A pair $(\hat{x},\hat{y})$ is called a Henig efficient element of (VP) if there exists $\varepsilon\in(0,\delta)$ such that

$$\bigl(F(\hat{E})-\hat{y}\bigr)\cap \bigl(-\operatorname{intcone} (\varepsilon U+B ) \bigr)=\emptyset, $$

where $\delta:=\inf\{\|b\|:b\in B\}$, $F(\hat{E})=\bigcup_{x\in\hat {E}}F(x)$, and *U* is the closed unit ball of *Y*.

### Definition 4.2

See [10]

The interior tangent cone $\operatorname{IT}(S,\bar{y})$ of *S* at *ȳ* is the set of all $y\in Y$ such that for any $t_{n}\rightarrow0^{+}$ and $y_{n}\rightarrow y$, we have $\bar{y}+t_{n}y_{n}\in S$.

### Remark 4.1

See [10]

If $S\subset Y$ is convex, $\bar{y}\in S$, and $\operatorname{int}S\neq\emptyset$, then

$$\operatorname{IT}(S,\bar{y})=\operatorname{IT}(\operatorname{int}S,\bar{y})= \operatorname {intcone}(S-\bar{y}). $$

### Theorem 4.1

*Suppose that* $(\hat{x},\hat{y})$ *is a Henig efficient element of* (VP), $\hat{z}\in G(\hat{x})\cap(-D)$, $(\hat{u},\hat{v},\hat{w})\in X\times (-C)\times(-D)$, *F* *is second*-*order lower radial composed derivable at* $(\hat{x},\hat{y})$ *in the direction* $(\hat{u},\hat{v})$, *and* *G* *is second*-*order radial composed derivable at* $(\hat{x},\hat{z})$ *in the direction* $(\hat{u},\hat{w})$. *Then there exists* $\hat{\varepsilon}\in(0,\delta)$ *such that*

4.1 $$ \bigl(R_{l}^{\prime\prime}F(\hat{x},\hat{y},\hat{u},\hat {v}) (x),R^{\prime\prime}G(\hat{x},\hat{z}, \hat{u},\hat{w}) (x) \bigr)\cap \bigl( \bigl(-\operatorname{intcone} (\hat{\varepsilon} U+B ) \bigr)\times ( - \operatorname {int}D ) \bigr)=\emptyset $$

*for all* $x\in\operatorname{dom}R_{l}^{\prime\prime}F(\hat{x},\hat {y},\hat{u},\hat{v})\cap\operatorname{dom}R^{\prime\prime}G(\hat {x},\hat{z},\hat{u},\hat{w})$.

### Proof

Since $(\hat{x},\hat{y})$ is a Henig efficient element of (VP), there exists a number $\varepsilon_{0}\in(0,\delta)$ such that

4.2 $$ \bigl(F(\hat{E})-\hat{y}\bigr)\cap \bigl(-\operatorname{intcone} (\varepsilon _{0} U+B ) \bigr)=\emptyset. $$

On the contrary, suppose that (4.1) does not hold. Then there exist $\bar{x}\in\operatorname{dom}R_{l}^{\prime\prime}F(\hat{x},\hat{y}, \hat {u},\hat{v})\cap\operatorname{dom}R^{\prime\prime}G(\hat{x},\hat {z},\hat{u},\hat{w})$, $\bar{y}\in R_{l}^{\prime\prime}F(\hat{x},\hat{y},\hat{u},\hat{v})(\bar {x})$, and $\bar{z}\in R^{\prime\prime}G(\hat{x},\hat{z},\hat{u},\hat {w})(\bar{x})$ such that

4.3 $$ \bar{y}\in-\operatorname{intcone} (\varepsilon_{0} U+B ) $$

and

4.4 $$ \bar{z}\in-\operatorname{int}D. $$

From $\bar{z}\in R^{\prime\prime}G(\hat{x},\hat{z},\hat{u},\hat{w})(\bar {x})$ it follows that

$$(\bar{x},\bar{z})\in\operatorname{graph}R^{\prime\prime}G(\hat{x},\hat {z}, \hat{u},\hat{w})=R\bigl(R\bigl(\operatorname{epi}G,(\hat{x},\hat{z})\bigr),(\hat {u},\hat{w})\bigr). $$

Hence, there exist $t_{n}>0$ and $(u_{n},w_{n})\in R(\operatorname {epi}G,(\hat{x},\hat{z}))$ such that

4.5 $$ t_{n}\bigl((u_{n},w_{n})-(\hat{u},\hat{w}) \bigr)\rightarrow(\bar{x},\bar {z}). $$

From (4.4) it follows that there exists $N_{1}\in N$ such that

$$t_{n} (w_{n}-\hat{w})\in-\operatorname{int}D,\quad \forall n>N_{1}. $$

Since $-\operatorname{int}D$ is a cone, we obtain

$$w_{n}-\hat{w}\in-\operatorname{int}D,\quad \forall n>N_{1}. $$

Since $\hat{w}\in-D$ and −*D* is a convex cone, it follows that

4.6 $$ w_{n}\in-\operatorname{int}D-D=-\operatorname{int}D,\quad \forall n>N_{1}. $$

Since $(u_{n},w_{n})\in R(\operatorname{epi}G,(\hat{x},\hat{z}))$, there exist sequences $t_{n}^{k}>0$ and $(x_{n}^{k},z_{n}^{k})\in\operatorname{epi}G$ such that

4.7 $$ t_{n}^{k} \bigl(\bigl(x_{n}^{k},z_{n}^{k} \bigr)-(\hat{x},\hat{z}) \bigr)\rightarrow(u_{n},w_{n}), \quad k\rightarrow+\infty. $$

It follows from (4.6) that there exists $K_{1}(n)\in N$ such that

$$t_{n}^{k}\bigl(z_{n}^{k}-\hat{z}\bigr) \in-\operatorname{int}D, \quad \forall n>N_{1}, \forall k>K_{1}(n). $$

Since $-\operatorname{int}D$ is a cone, we obtain

$$z_{n}^{k}-\hat{z}\in-\operatorname{int}D,\quad \forall n>N_{1}, \forall k>K_{1}(n). $$

Since $\hat{z}\in-D$ and −*D* is a convex cone, it follows that

$$z_{n}^{k}\in-\operatorname{int}D-D=-\operatorname{int}D, \quad \forall n>N_{1}, \forall k>K_{1}(n). $$

Since $(x_{n}^{k},z_{n}^{k})\in\operatorname{epi}G$, we obtain $z_{n}^{k}\in G(x_{n}^{k})+D$. Hence, there exists $\bar{z}_{n}^{k}\in G(x_{n}^{k})$ such that $z_{n}^{k}\in\bar{z}_{n}^{k}+D$. Consequently,

$$\bar{z}_{n}^{k}\in z_{n}^{k}-D\subset- \operatorname {int}D-D=-\operatorname{int}D. $$

Thus, $G(x_{n}^{k})\cap(-D)\neq\emptyset$, that is, $x_{n}^{k}\in\hat{E}$. It follows from (4.7) that $t_{n}^{k}(x_{n}^{k}-\hat{x})\rightarrow u_{n}$ as $k\rightarrow\infty$, and hence, $u_{n}\in R(\hat{E},\hat{x})$. It follows from (4.5) that $t_{n}(u_{n}-\hat{u})\rightarrow\bar{x}$, and hence, $\bar{x}\in R(R(\hat{E},\hat{x}),\hat{u})$. By Proposition 3.1, since $\bar{y}\in R_{l}^{\prime\prime}F(\hat{x},\hat {y},\hat{u},\hat{v})(\bar{x})$, we conclude that

$$\bar{y}\in R_{l}^{\prime\prime}F(\hat{x},\hat{y},\hat{u},\hat {v}) \bigl(R\bigl(R(\hat{E},\hat{x}),\hat{u}\bigr)\bigr)\subset\operatorname{clcone} \bigl(\operatorname{clcone}\bigl(F(\hat{E})+C-\hat{y}\bigr)-\hat{v} \bigr). $$

From (4.3) it follows that

$$\operatorname{clcone} \bigl(\operatorname{clcone}\bigl(F(\hat{E})+C-\hat {y} \bigr)-\hat{v} \bigr)\cap \bigl(-\operatorname{intcone} (\varepsilon _{0} U+B ) \bigr)\neq\emptyset. $$

Since $-\operatorname{intcone} (\varepsilon_{0} U+B )$ is open, we obtain

$$\operatorname{cone} \bigl(\operatorname{clcone}\bigl(F(\hat{E})+C-\hat {y} \bigr)-\hat{v} \bigr)\cap \bigl(-\operatorname{intcone} (\varepsilon _{0} U+B ) \bigr)\neq\emptyset. $$

Since $\operatorname{cone}(\varepsilon_{0} U+B)$ is a pointed cone, it follows that

$$\bigl(\operatorname{clcone}\bigl(F(\hat{E})+C-\hat{y}\bigr)-\hat{v} \bigr)\cap \bigl(-\operatorname{intcone} (\varepsilon_{0} U+B ) \bigr)\neq \emptyset, $$

and thus,

$$\operatorname{clcone}\bigl(F(\hat{E})+C-\hat{y}\bigr)\cap \bigl(\hat {v}- \operatorname{intcone} (\varepsilon_{0} U+B ) \bigr)\neq \emptyset. $$

It follows from $\hat{v}\in-C\subset-\operatorname{cone}(\varepsilon _{0} U+B)$ that

$$\begin{aligned} \hat{v}-\operatorname{int} \bigl(\operatorname {cone}(\varepsilon_{0} U+B) \bigr)&\subset-\operatorname {cone}(\varepsilon_{0} U+B)- \operatorname{int} \bigl(\operatorname {cone}(\varepsilon_{0} U+B) \bigr) \\ & \subset-\operatorname{intcone} (\varepsilon_{0} U+B ). \end{aligned}$$

Consequently,

$$\operatorname{clcone}\bigl(F(\hat{E})+C-\hat{y}\bigr)\cap \bigl(-\operatorname {intcone} (\varepsilon_{0} U+B ) \bigr)\neq\emptyset. $$

In the similar way, we conclude that

$$\bigl(F(\hat{E})+C-\hat{y}\bigr)\cap \bigl(-\operatorname{intcone} ( \varepsilon_{0} U+B ) \bigr)\neq\emptyset. $$

Since $C\subset\operatorname{cone}(\varepsilon_{0} U+B)$ and $\operatorname{cone}(\varepsilon_{0} U+B)$ is a point cone, we obtain

$$\bigl(F(\hat{E})-\hat{y}\bigr)\cap \bigl(-\operatorname{intcone} (\varepsilon _{0} U+B ) \bigr)\neq\emptyset. $$

This is a contradiction to (4.2). The proof is completed. □

### Corollary 4.1

*Suppose that* $(\hat{x},\hat{y})$ *is a Henig efficient element of* (VP), $\hat{z}\in G(\hat{x})\cap(-D)$, $(\hat{u},\hat{v},\hat{w})\in X\times (-C)\times(-D)$, *F* *is second*-*order lower radial composed derivable at* $(\hat{x},\hat{y})$ *in the direction* $(\hat{u},\hat{v})$, *and* *G* *is second*-*order lower radial composed derivable at* $(\hat{x},\hat{z})$ *in the direction* $(\hat{u},\hat{w})$. *Then there exists a number* $\hat{\varepsilon}\in(0,\delta)$ *such that*

$$\bigl(R_{l}^{\prime\prime}F(\hat{x},\hat{y},\hat{u},\hat {v}) (x),R_{l}^{\prime\prime}G(\hat{x},\hat{z}, \hat{u},\hat{w}) (x) \bigr) \cap \bigl( \bigl(-\operatorname{intcone} (\hat{\varepsilon} U+B ) \bigr)\times (- \operatorname {int}D ) \bigr)=\emptyset $$

*for all* $x\in\operatorname{dom}R_{l}^{\prime\prime}F(\hat{x},\hat {y},\hat{u},\hat{v})\cap\operatorname{dom}R_{l}^{\prime\prime}G(\hat {x},\hat{z},\hat{u},\hat{w})$.

### Proof

The proof follows directly from Theorem 4.1 and Remark 2.3(ii). □

### Corollary 4.2

*Suppose that* $(\hat{x},\hat{y})$ *is a Henig efficient element of* (VP), $\hat{z}\in G(\hat{x})\cap(-D)$, $(\hat{u},\hat{v},\hat{w})\in X\times(-C)\times(-D)$, *C* *has a convex base* *B*, *F* *is second*-*order lower radial composed derivable at* $(\hat{x},\hat{y})$ *in the direction* $(\hat{u},\hat{v})$, *and* *G* *is second*-*order lower radial composed derivable at* $(\hat{x},\hat{z})$ *in the direction* $(\hat{u},\hat{w})$. *Then there exists a number* $\hat{\varepsilon}\in(0,\delta)$ *such that*

$$\bigl(R_{l}^{\prime\prime}F(\hat{x},\hat{y},\hat{u},\hat {v}) (x),R_{l}^{\prime\prime}G(\hat{x},\hat{z}, \hat{u},\hat{w}) (x) \bigr) \cap \bigl(\operatorname{IT} \bigl(-\operatorname {intcone} (\hat{\varepsilon} U+B ),-\hat{v} \bigr)\times (-\operatorname{int}D ) \bigr)=\emptyset $$

*for all* $x\in\operatorname{dom}R_{l}^{\prime\prime}F(\hat{x},\hat {y},\hat{u},\hat{v})\cap\operatorname{dom}R_{l}^{\prime\prime}G(\hat {x},\hat{z},\hat{u},\hat{w})$.

### Proof

$$\begin{aligned} \operatorname{IT} \bigl(-\operatorname{intcone} (\hat{\varepsilon } U+B ),- \hat{v} \bigr) =&\operatorname{intcone} \bigl(-\operatorname{intcone} (\hat{ \varepsilon} U+B )+\hat{v} \bigr) \\ \subset&-C-\operatorname{intcone} (\hat{\varepsilon} U+B ) \\ \subset&-\operatorname{cone}(\hat{\varepsilon} U+B)-\operatorname {intcone} ( \hat{\varepsilon} U+B ) \\ \subset&-\operatorname{intcone} (\hat{\varepsilon} U+B ). \end{aligned}$$

 □

We provide the following example to explain Theorem 4.1 and Corollaries 4.1 and 4.2.

### Example 4.1

Let *R* be the set of real numbers, $X=Y=Z=R$, $C=D=\{t:t\geq0\}$, $B=\{1\}$. Define the set-valued maps $F:X\rightarrow2^{Y}$ and $G:X\rightarrow2^{Z}$ by

$$F(x)=G(x)=\left \{ \textstyle\begin{array}{l@{\quad}l} \{y:y\geq0\}& \mbox{if } x\geq0, \\ \{y:y\geq x^{2}\} & \mbox{otherwise}. \end{array}\displaystyle \right . $$

Let $(\hat{x},\hat{y})=(0,0)$, $(\hat{u},\hat{v},\hat{w})=(1,0,0)\in X\times(-C)\times(-D)$, $\varepsilon=\frac{1}{2}$. A direct calculation gives

$$\begin{aligned}& \hat{z}\in G(0)\cap(-D)=\{0\}, \\& R_{l}\bigl(\operatorname{epi}F,(0,0)\bigr)=R_{l}\bigl( \operatorname{epi}G,(0,0)\bigr)=\bigl\{ (x,y):x> 0,y\geq0\bigr\} , \\& R\bigl(\operatorname{epi}G,(0,0)\bigr)=\bigl\{ (x,y):x\in R,y\geq0\bigr\} , \\& R_{l} \bigl(R_{l}\bigl(\operatorname{epi}F,(0,0) \bigr),(1,0) \bigr)=R_{l} \bigl(R_{l}\bigl( \operatorname{epi}G,(0,0)\bigr),(1,0) \bigr)=\bigl\{ (x,y):x> 0,y\geq0\bigr\} , \\& R \bigl(R\bigl(\operatorname{epi}G,(0,0)\bigr),(1,0) \bigr)=\bigl\{ (x,y):x\in R,y\geq 0\bigr\} , \\& R_{l}^{\prime\prime}F(0,0,1,0) (x)=R_{l}^{\prime\prime }G(0,0,1,0) (x)=\left \{ \textstyle\begin{array}{l@{\quad}l} \{y:y\geq0\}& \mbox{if } x>0, \\ \emptyset& \mbox{otherwise}, \end{array}\displaystyle \right . \\& R^{\prime\prime}G(0,0,1,0) (x)=[0,+\infty),\quad x\in R, \\& \operatorname{IT} \bigl(-\operatorname{intcone} (\varepsilon U+B ),-\hat {v} \bigr)=-\operatorname{intcone} (\varepsilon U+B )=(-\infty,0). \end{aligned}$$

Then, the inclusions of Theorem 4.1 and Corollaries 4.1 and 4.2 are true.

### Theorem 4.2

*Suppose that* $(\hat{x},\hat{y})$ *is a Henig efficient element of* (VP), $\hat{z}\in G(\hat{x})\cap(-D)$, $(\hat{u},\hat{v},\hat{w})\in X\times (-C)\times(-D)$, *F* *is second*-*order lower radial derivable at* $(\hat{x},\hat{y})$ *in the direction* $(\hat{u},\hat{v})$, *and* *G* *is second*-*order radial derivable at* $(\hat{x},\hat{z})$ *in the direction* $(\hat{u},\hat{w})$. *Then there exists a number* $\hat{\varepsilon}\in(0,\delta)$ *such that*

4.8 $$ \bigl(R_{l}^{2}F(\hat{x},\hat{y},\hat{u},\hat{v}) (x),R^{2}G(\hat{x},\hat{z}, \hat{u},\hat{w}) (x) \bigr)\cap \bigl( \bigl(-\operatorname{intcone} (\hat{\varepsilon} U+B ) \bigr)\times (- \operatorname {int}D ) \bigr)=\emptyset $$

*for all* $x\in\operatorname{dom}R_{l}^{2}F(\hat{x},\hat{y},\hat{u},\hat {v})\cap\operatorname{dom}R^{2}G(\hat{x},\hat{z},\hat{u},\hat{w})$.

### Proof

On the contrary, suppose that (4.8) does not hold. Then, for any $\varepsilon\in(0,\delta)$, there exist $\bar{x}\in \operatorname{dom}R_{l}^{2}F(\hat{x},\hat{y}, \hat{u},\hat{v})\cap \operatorname{dom}R^{2}G(\hat{x},\hat{z},\hat{u},\hat{w})$, $\bar{y}\in R_{l}^{2}F(\hat{x},\hat{y},\hat{u},\hat{v})(\bar{x})$, and $\bar{z}\in R^{2}G(\hat{x},\hat{z},\hat{u},\hat{w})(\bar{x})$ such that

4.9 $$ \bar{y}\in-\operatorname{intcone} (\varepsilon U+B ) $$

and

4.10 $$ \bar{z}\in-\operatorname{int}D. $$

From $\bar{z}\in R^{2}G(\hat{x},\hat{z},\hat{u},\hat{w})(\bar{x})$ it follows that

$${(\bar{x},\bar{z})}\in \operatorname {graph}R^{2}G(\hat{x},\hat{z}, \hat{u},\hat{w}) =R^{2}\bigl(\operatorname{epi}G,(\hat{x},\hat{z}),( \hat{u},\hat{w})\bigr). $$

Hence, there exist $t_{n}>0$, $x_{n}\rightarrow\bar{x}$, and $z_{n}\rightarrow\bar{z}$ such that

$$\biggl(\hat{x}+t_{n}\hat{u}+\frac{1}{2}t_{n}^{2}x_{n}, \hat{z}+t_{n}\hat {w}+\frac{1}{2}t_{n}^{2}z_{n} \biggr)\in\operatorname{epi}G. $$

Thus,

4.11 $$ \hat{z}+t_{n}\hat{w}+\frac{1}{2}t_{n}^{2}z_{n} \in G\biggl(\hat{x}+t_{n}\hat {u}+\frac{1}{2}t_{n}^{2}x_{n} \biggr)+D. $$

The set of positive integers is denoted by *N*. From (4.10) and $z_{n}\rightarrow\bar{z}$ it follows that there exists $N_{1}\in N$ such that

$$z_{n}\in-\operatorname{int}D,\quad \forall n>N_{1}. $$

Since $-\operatorname{int}D$ and −*D* are convex cones, we obtain

4.12 $$ \hat{z}+t_{n}\hat{w}+\frac{1}{2}t_{n}^{2}z_{n} \in-D-D-\operatorname {int}D= -\operatorname{int}D, \quad \forall n>N_{1}. $$

It follows from (4.11) that there exists $\tilde{z}_{n}\in G(\hat {x}+t_{n}\hat{u}+\frac{1}{2}t_{n}^{2}x_{n})$ such that

$$\hat{z}+t_{n}\hat{w}+\frac{1}{2}t_{n}^{2}z_{n} \in\{\tilde{z}_{n}\}+D. $$

Since (4.12) and *D* is a convex cone, we obtain

$$\tilde{z}_{n}\in\biggl\{ \hat{z}+t_{n}\hat{w}+ \frac{1}{2}t_{n}^{2}z_{n}\biggr\} -D\subset- \operatorname{int}D-D=-\operatorname{int}D\subset-D. $$

Thus,

$$G\biggl(\hat{x}+t_{n}\hat{u}+\frac{1}{2}t_{n}^{2}x_{n} \biggr)\cap(-D)\neq\emptyset, $$

that is,

$$\hat{x}+t_{n}\hat{u}+\frac{1}{2}t_{n}^{2}x_{n} \in\hat{E}. $$

From $t_{n}>0$ and $x_{n}\rightarrow\bar{x}$ it follows that $\bar {x}\in R^{2}(\hat{E},\hat{x},\hat{u})$. By Proposition 3.2 and $\bar{y}\in R_{l}^{2}F(\hat{x},\hat{y},\hat {u},\hat{v})(\bar{x})$ we obtain

$$\bar{y}\in R_{l}^{2}F(\hat{x},\hat{y},\hat{u},\hat{v}) \bigl(R^{2}(\hat {E},\hat{x},\hat{u}) \bigr)\subset\operatorname{clcone} \bigl(\operatorname{cone}\bigl(F(\hat{E})+C-\hat{y}\bigr)-\hat{v} \bigr). $$

It follows from (4.9) that

$$\operatorname{clcone} \bigl(\operatorname{cone}\bigl(F(\hat{E})+C-\hat {y} \bigr)-\hat{v} \bigr)\cap \bigl(-\operatorname{intcone} (\varepsilon U+B ) \bigr) \neq\emptyset. $$

Since $-\operatorname{intcone} (\varepsilon U+B )$ is open, we obtain

$$\operatorname{cone} \bigl(\operatorname{cone}\bigl(F(\hat{\hat{E}})+C-\hat {y} \bigr)-\hat{v} \bigr)\cap \bigl(-\operatorname{intcone} (\varepsilon U+B ) \bigr) \neq\emptyset. $$

Since $\operatorname{cone}(\varepsilon U+B)$ is a pointed cone, it follows that

$$\bigl(\operatorname{cone}\bigl(F(\hat{E})+C-\hat{y}\bigr)-\hat{v} \bigr)\cap \bigl(-\operatorname{intcone} (\varepsilon U+B ) \bigr)\neq \emptyset, $$

and thus,

$$\operatorname{cone}\bigl(F(\hat{E})+C-\hat{y}\bigr)\cap \bigl(\hat {v}- \operatorname{intcone} (\varepsilon U+B ) \bigr)\neq \emptyset. $$

It follows from $\hat{v}\in-C\subset-\operatorname{cone}(\varepsilon U+B)$ that

$$\begin{aligned} \hat{v}-\operatorname{int} \bigl(\operatorname {cone}(\varepsilon U+B) \bigr)& \subset-\operatorname{cone}(\varepsilon U+B)-\operatorname{int} \bigl( \operatorname{cone}(\varepsilon U+B) \bigr) \\ & \subset-\operatorname{intcone} (\varepsilon U+B ). \end{aligned}$$

Consequently,

$$\operatorname{cone}\bigl(F(\hat{E})+C-\hat{y}\bigr)\cap \bigl(-\operatorname {intcone} (\varepsilon U+B ) \bigr)\neq\emptyset. $$

In a similar way, we conclude that

$$\bigl(F(\hat{E})+C-\hat{y}\bigr)\cap \bigl(-\operatorname{intcone} (\varepsilon U+B ) \bigr)\neq\emptyset. $$

Since $C\subset\operatorname{cone}(\varepsilon U+B)$ and $\operatorname {cone}(\varepsilon U+B)$ is a pointed cone, we obtain

$$\bigl(F(\hat{E})-\hat{y}\bigr)\cap \bigl(-\operatorname{intcone} (\varepsilon U+B ) \bigr)\neq\emptyset. $$

This is a contradiction to the assumption that $(\hat{x},\hat{y})$ is a Henig minimizer of (VP). □

### Corollary 4.3

*Suppose that* $(\hat{x},\hat{y})$ *is a Henig efficient element of* (VP), $\hat{z}\in G(\hat{x})\cap(-D)$, $(\hat{u},\hat{v},\hat{w})\in X\times (-C)\times(-D)$, *F* *is second*-*order lower radial derivable at* $(\hat{x},\hat{y})$ *in the direction* $(\hat{u},\hat{v})$, *and* *G* *is second*-*order lower radial derivable at* $(\hat{x},\hat{z})$ *in the direction* $(\hat{u},\hat{w})$. *Then there exists a number* $\hat{\varepsilon}\in(0,\delta)$ *such that*

$$\bigl(R_{l}^{2}F(\hat{x},\hat{y},\hat{u},\hat{v}) (x),R_{l}^{2}G(\hat {x},\hat{z}, \hat{u},\hat{w}) (x) \bigr) \cap \bigl( \bigl(-\operatorname{intcone} (\hat{\varepsilon} U+B ) \bigr)\times (- \operatorname {int}D ) \bigr)=\emptyset $$

*for all* $x\in\operatorname{dom}R_{l}^{2}F(\hat{x},\hat{y},\hat{u},\hat {v})\cap\operatorname{dom}R_{l}^{2}G(\hat{x},\hat{z},\hat{u},\hat {w})$.

### Proof

The proof follows immediately from Theorem 4.2 and Remark 2.3(ii). □

### Corollary 4.4

*Suppose that* $(\hat{x},\hat{y})$ *is a Henig efficient element of* (VP), $\hat{z}\in G(\hat{x})\cap(-D)$, $(\hat{u},\hat{v},\hat{w})\in X\times(-C)\times(-D)$, *B* *is a base of* *C*, *F* *is second*-*order lower radial derivable at* $(\hat{x},\hat{y})$ *in the direction* $(\hat{u},\hat{v})$, *and* *G* *is second*-*order lower radial derivable at* $(\hat{x},\hat{z})$ *in the direction* $(\hat{u},\hat{w})$. *Then there exists a number* $\hat{\varepsilon}\in(0,\delta)$ *such that*

$$\bigl(R_{l}^{2}F(\hat{x},\hat{y},\hat{u},\hat{v}) (x),R_{l}^{2}G(\hat {x},\hat{z}, \hat{u},\hat{w}) (x) \bigr) \cap \bigl(\operatorname{IT} \bigl(-\operatorname {intcone} (\hat{\varepsilon} U+B ),-\hat{v} \bigr)\times (-\operatorname{int}D ) \bigr)=\emptyset $$

*for all* $x\in\operatorname{dom}R_{l}^{2}F(\hat{x},\hat{y},\hat{u},\hat {v})\cap\operatorname{dom}R_{l}^{2}G(\hat{x},\hat{z},\hat{u},\hat {w})$.

### Proof

It is similar to the proof of Corollary 4.2. □

We give the following example to illustrate Theorem 4.2 and Corollaries 4.3 and 4.4.

### Example 4.2

Let *R* be the set of real numbers, $X=Y=Z=R$, $C=D=\{t:t\geq0\}$, and $B=\{1\}$. Define the set-valued maps $F:X\rightarrow2^{Y}$ and $G:X\rightarrow2^{Z}$ by

$$\begin{aligned}& F(x)=\{y:y\geq0\},\quad x\in R, \\& G(x)=\{y:y\geq x\},\quad x\in R. \end{aligned}$$

Let $(\hat{x},\hat{y})=(0,0)$, $(\hat{u},\hat{v},\hat{w})=(-1,0,-1)$, and $\varepsilon=\frac{1}{2}$. A direct calculation gives

$$\begin{aligned}& \hat{z}\in G(0)\cap(-D)=\{0\}, \\& R_{l}^{2}\bigl(\operatorname{epi}F,(0,0),(-1,0)\bigr)=\bigl\{ (x,y):x\in R,y\geq0\bigr\} , \\& R^{2}\bigl(\operatorname{epi}G,(0,0),(-1,-1)\bigr)=\bigl\{ (x,y):x\in R,y\geq x\bigr\} , \\& R_{l}^{2}\bigl(\operatorname{epi}G,(0,0),(-1,-1)\bigr)= \bigl\{ (x,y):x\in R,y\geq x\bigr\} , \\& R_{l}^{2}F(0,0,-1,0) (x)=\{y:y\geq0\},\quad x\in R, \\& R_{l}^{2}G(0,0,-1,-1) (x)=R^{2}G(0,0,-1,-1) (x)= \{y:y\geq x\},\quad x\in R, \\& \operatorname{IT} \bigl(-\operatorname{intcone} (\varepsilon U+B ),-\hat {v} \bigr)=-\operatorname{intcone} (\varepsilon U+B )=(-\infty,0). \end{aligned}$$

Then, the inclusions of Theorem 4.2 and Corollaries 4.3 and 4.4 are true.

Let us recall that the upper (inferior) limit in the sense of Painlevé-Kuratowski of a set-valued map $\Phi:X\rightarrow2^{Y} $ is defined as $\limsup_{u\rightarrow\bar{u}}\Phi(u):=\{y\in Y:\exists u_{n}\rightarrow\bar{u}, \exists y_{n}\in\Phi(u_{n}) \mbox{ such that } y_{n}\rightarrow y\}$ and $\liminf_{u\rightarrow\bar{u}}\Phi(u):=\{y\in Y:\forall u_{n}\rightarrow\bar{u}, \exists y_{n}\in\Phi(u_{n}) \mbox{ such that}\ y_{n}\rightarrow y\}$. If $f:X\rightarrow Y$ is Fréchet differentiable at $\hat{x}\in X$, its Fréchet derivative is denoted by $f^{\prime}(\hat{x})$.

The profile map of *F* is the set-valued map $F_{+}:X\rightarrow 2^{Y}$ defined by $F_{+}(x)=F(x)+C$, $x\in\operatorname{dom} F$.

In what follows, we consider vector optimization.

Let $f:X\rightarrow Y$, $g:X\rightarrow Z$.

Consider the following vector optimization:

$$\begin{aligned} \begin{aligned} (\mathrm{P})&\quad \min f(x), \\ &\qquad \mbox{s.t. }g(x)\in-D,\quad x\in X. \end{aligned} \end{aligned}$$

Similarly to Definition 4.3 in [18], we introduce the following second-order generalized lower (upper) directional derivative for vector-valued functions.

### Definition 4.3

Let $f:X\rightarrow Y$ be Fréchet differentiable at *x̂*, and $\hat{u},x\in X$. The parabolic second-order generalized lower directional derivative of *x̂* in the direction $(\hat{u},x)$ is

$$\tilde{D}_{l}^{2}f(\hat{x},\hat{u}) (x):=\liminf _{t>0, x^{\prime }\rightarrow x}\frac{f(\hat{x}+t\hat{u}+\frac{1}{2}t^{2}x^{\prime })-f(\hat{x})-tf^{\prime}(\hat{x})\hat{u}}{\frac{1}{2}t^{2}}. $$

### Remark 4.2

When the set-valued map *F* becomes to a vector-valued function *f*, which is Fréchet differentiable at *x̂*, letting $\hat{v}:=f^{\prime}(\hat{x})\hat{u}$, we have

$$R_{l}^{2}F(\hat{x},\hat{y},\hat{u},\hat{v}) (x)=\tilde {D}_{l}^{2}f_{+}(\hat{x},\hat{u}) (x)=\liminf _{t>0, x^{\prime }\rightarrow x} \frac{f_{+}(\hat{x}+t\hat{u}+\frac{1}{2}t^{2}x^{\prime })-f_{+}(\hat{x})-tf^{\prime}(\hat{x})\hat{u}}{\frac{1}{2}t^{2}}. $$

### Definition 4.4

Let $f:X\rightarrow Y$ be Fréchet differentiable at *x̂*, and $\hat{u},x\in X$. The parabolic second-order generalized upper directional derivative of *x̂* in the direction $(\hat{u},x)$ is

$$\tilde{D}^{2}f(\hat{x},\hat{u}) (x):=\limsup_{t>0, x^{\prime}\rightarrow x} \frac{f(\hat{x}+t\hat{u}+\frac{1}{2}t^{2}x^{\prime})-f(\hat {x})-tf^{\prime}(\hat{x})\hat{u}}{\frac{1}{2}t^{2}}. $$

### Remark 4.3

When the set-valued map *F* becomes to a vector-valued function *f*, which is Fréchet differentiable at *x̂*, letting $\hat{v}:=f^{\prime}(\hat{x})\hat{u}$, we have

$$R^{2}F(\hat{x},\hat{y},\hat{u},\hat{v}) (x)=\tilde{D}^{2}f_{+}( \hat {x},\hat{u}) (x)=\limsup_{t>0, x^{\prime}\rightarrow x} \frac{f_{+}(\hat {x}+t\hat{u}+\frac{1}{2}t^{2}x^{\prime})-f_{+}(\hat{x})-tf^{\prime}(\hat {x})\hat{u}}{\frac{1}{2}t^{2}}. $$

### Corollary 4.5

*Suppose that* $(\hat{x},\hat{y})$ *is a Henig efficient element of* (P) *and* $g(\hat{x})\in-D$. *Then there exists a number* $\hat{\varepsilon}\in (0,\delta)$ *such that*

$$\bigl(\tilde{D}_{l}^{2}f_{+}(\hat{x},\hat{u}) (x),\tilde{D}^{2}g_{+}(\hat {x},\hat{u}) (x) \bigr)\cap \bigl( \bigl(-\operatorname{intcone} (\hat {\varepsilon} U+B ) \bigr)\times (- \operatorname{int}D ) \bigr)=\emptyset $$

*for any* $x\in\operatorname{dom}\tilde{D}_{l}^{2}f_{+}(\hat{x},\hat {u})\cap\operatorname{dom}\tilde{D}^{2}g_{+}(\hat{x},\hat{u})$.

### Proof

The proof follows immediately from Theorem 4.2 and Remarks 4.2 and 4.3. □

## Conclusions

In this paper, we introduced some new kinds of lower radial tangent cone, second-order lower radial tangent set, and second-order radial tangent set. By virtue of these concepts, second-order radial composed tangent derivative, second-order radial tangent derivative, second-order lower radial composed tangent derivative, and second-order lower radial tangent derivative for a set-valued map are introduced. Compared with the second-order composed contingent derivative $D^{\prime\prime}F(\hat{x},\hat{y},\hat{u},\hat{v})$ introduced in [4, 15], the second-order contingent derivative $D^{2}F(\hat{x},\hat{y},\hat{u},\hat{v})$, second-order radial composed tangent derivative $R^{\prime\prime}F(\hat{x},\hat{y},\hat{u},\hat{v})$, and second-order radial tangent derivative $R^{2}F(\hat{x},\hat{y},\hat {u},\hat{v})$, second-order lower radial composed tangent derivative $R_{l}^{\prime\prime}F(\hat{x},\hat{y},\hat{u},\hat{v})$, and second-order lower radial tangent derivative $R_{l}^{2}F(\hat{x},\hat {y},\hat{u},\hat{v})$ have nice properties:

$$R_{l}^{\prime\prime}F(\hat{x},\hat{y},\hat{u},\hat{v}) \bigl(R \bigl(R(E,\hat {x}),\hat{u}\bigr) \bigr)\subset\operatorname{clcone} \bigl( \operatorname{clcone}\bigl(F(E)+C-\hat{y}\bigr)-\hat{v} \bigr) $$

and

$$R_{l}^{2}F(\hat{x},\hat{y},\hat{u},\hat{v}) \bigl(R^{2}(E,\hat{x},\hat {u}) \bigr)\subset\operatorname{clcone} \bigl(\operatorname{cone}\bigl(F(E)+C-\hat{y}\bigr)-\hat{v} \bigr), $$

which are demonstrated in Propositions 3.1 and 3.2. Just applying these properties, we established second-order necessary optimality conditions for a point pair to be a Henig efficient element of a set-valued optimization problem where the second-order tangent derivatives of the objective function and constraint function are separated.
